# COMMUNITY-DWELLING OLDER ADULTS’ PERCEPTIONS OF SMART HOME SURVEILLANCE: AN INTEGRATIVE REVIEW

**DOI:** 10.1093/geroni/igac059.2772

**Published:** 2022-12-20

**Authors:** Shehroz Khan, Jessica Campbell, Jacob Buchan, Andria Bianchi, Jesse Hoey, Charlene Chu

**Affiliations:** Toronto Rehabilitation Institute, Toronto, Ontario, Canada; University of Victoria, Victoria, British Columbia, Canada; University of Toronto, Toronto, Ontario, Canada; Toronto Rehabilitation Institute, Toronto, Ontario, Canada; University of Waterloo, Waterloo, Ontario, Canada; University of Toronto, Toronto, Ontario, Canada

## Abstract

**Background:**

Many older adults wish to use smart homes for aging in place, health monitoring, and enhanced safety. However, concerns over privacy and security remain pressing. User perception studies can help to inform policy and design solutions. Aim: To explore community-dwelling older adults’ (50+) perceptions of smart home surveillance.

**Methods:**

As part of a larger scoping review of smart home user perception based on four non-mutually exclusive categories: privacy, safety, purpose of data collection, and risk, we found 68 results. 15 studies focused on older adults exclusively and were included in this review.

**Results:**

The included studies mainly focused on smart speakers, motion sensors, or home monitoring systems. 13 studies (87%) discussed user privacy concerns in terms of data collection and access. Nine studies (60%) reported that users were enthusiastic about the potential for home safety, improved health outcomes and independent living with smart homes. Seven risk awareness studies (47%) featured a range of perspectives on sharing sensitive information due to the possibility of data breaches and third-party misuse, with some reporting a willingness to trade privacy for enhanced safety. Finally, four studies (27%) explored user knowledge of data collection purposes. While many were uncertain of the details, users were generally more comfortable sharing smart home data with healthcare professionals than others.

**Conclusion:**

This review has helped us in creating a user perception survey that is currently in the fielding stage. Given Canada’s increasing aging population and technological advances, privacy regulators and designers should focus on older adults’ concerns.

